# Descriptors construction and application in catalytic site design

**DOI:** 10.1016/j.isci.2025.113080

**Published:** 2025-07-09

**Authors:** Yuqing Feng, Yushun Chen, Li Zheng, Xuetao Chen, Tan Li, Wenbo Zhao

**Affiliations:** 1 Faculty of Chemical Engineering, Kunming University of Science and Technology, Kunming 650500, China

**Keywords:** Chemistry, Applied chemistry, Materials science

## Abstract

Descriptors are quantitative or qualitative measures that capture key properties of a system. In catalysis, they are essential for understanding the relationship between a material’s structure and its function, facilitating the design and optimization of new catalytic materials and processes. Since the introduction of energy descriptors in the 1970s, various approaches, ranging from electronic properties to data-driven techniques, have been developed to construct these descriptors. This review first categorizes the different types of descriptors, including energy, electronic, and data-driven approaches. It then explores their applications in active site design and finally concludes with a discussion on the challenges and opportunities in advancing catalytic site design.

## Graphical abstract

**Figure:**
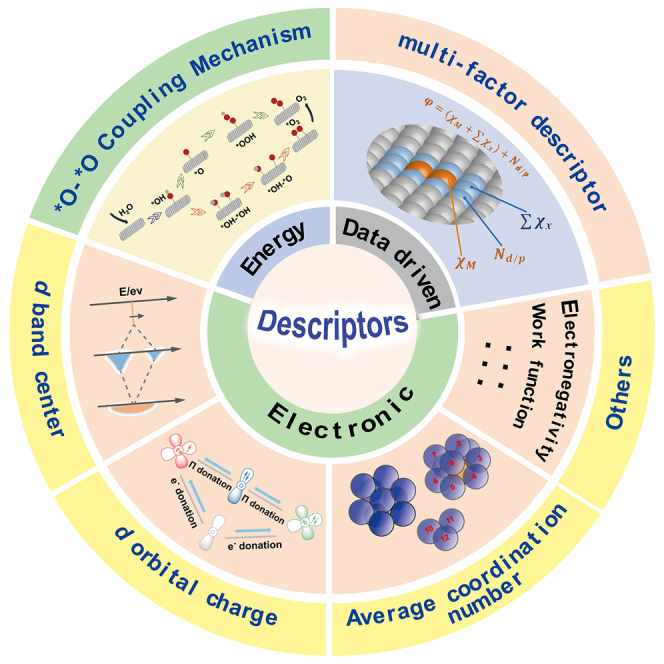

## Introduction

Descriptors are crucial tools for quantifying and characterizing the properties of active sites on catalyst surfaces, playing a key role in understanding and predicting catalytic processes. In the 1970s, Trasatti pioneered the use of descriptors in catalyst design by proposing the heat of hydrogen adsorption on different metals, combined with volcano plots, to describe the hydrogen evolution reaction.^1^ Initially, energy descriptors were used to relate surface reaction energies to catalyst activation energies. Over time, a range of energy descriptors has been developed to directly reflect the energy states of molecules or materials, enabling more accurate predictions of catalyst activity and reaction outcomes. However, although energy descriptors offer valuable insights, they have limitations. They provide limited information about the electronic structures of catalysts, which hampers their ability to explain specific electronic behaviors at the molecular level. In catalytic reactions, adsorption and transition state energies are often governed by scaling relationships, which complicate the use of energy descriptors to explain local molecular properties.^2^^,^^3^^,^^4^^,^^5^^,^^6^ Moreover, applying energy descriptors to large or complex systems can be computationally demanding and time-consuming. These challenges limit the practical use of energy descriptors in the design of efficient catalysts.

In the 1990s, Jens Nørskov and Bjørk Hammer introduced the *d*-band center theory for transition metal catalysts, demonstrating how the position of the *d*-band center influences the adsorption capacity of adsorbates on metal surfaces.^7^ This marked the first use of molecular *d*-orbital information from a microscopic perspective, providing crucial insights into catalyst activity and selectivity. Descriptors derived from the *d*-band center theory calculate the average energy of *d*-orbital levels, offering valuable information about electronic structure across different scales. These descriptors effectively capture the geometric properties of molecules and crystals while improving computational efficiency, helping to mitigate the limitations posed by scaling relationships. Despite these advantages, electronic descriptors face challenges. They do not always correlate well with experimentally measurable factors, such as electronegativity or atomic radius, and can struggle with managing large sets of experimental data. As catalytic systems grow in complexity, the ability of electronic descriptors to capture subtle electronic effects and the intricate details of the electronic structure becomes increasingly limited. In modern chemical and energy industries, descriptors serve as the core tools for enabling precision catalysis. By guiding atomic-scale design to enhance selectivity and efficiency while reducing precious-metal usage and pollution, they underpin sustainable processes such as green synthesis and wastewater treatment.^8^^,^^9^ Moreover, descriptors optimize the performance of key materials in fuel cells, water electrolysis, and related technologies, accelerating the commercial deployment of renewable energy solutions.^10^^,^^11^^,^^12^

In recent years, advances in computational methods and the integration of big data technologies have led to the increasing use of data-driven descriptors in catalytic site design. By integrating machine learning, high-throughput screening, and *in situ* characterization, descriptors will evolve into dynamic, intelligent tools, propelling catalytic materials from empirical design to a theory-driven industrial revolution. These descriptors enable precise predictions of catalytic performance, offering practical guidance for identifying high-performance catalysts. By incorporating key physicochemical properties, such as electronegativity and atomic radius, they establish mathematical relationships between catalyst structure and adsorption energy.^13^ This allows for rapid learning from experimental data, optimizing catalyst design and accelerating the development of new materials. The use of big data technology has further enhanced the power of data-driven descriptors,^14^^,^^15^^,^^16^^,^^17^^,^^18^^,^^19^^,^^20^ significantly improving prediction speed compared to traditional density functional theory (DFT) calculations and driving transformative progress in materials science.

**Figure 1:**
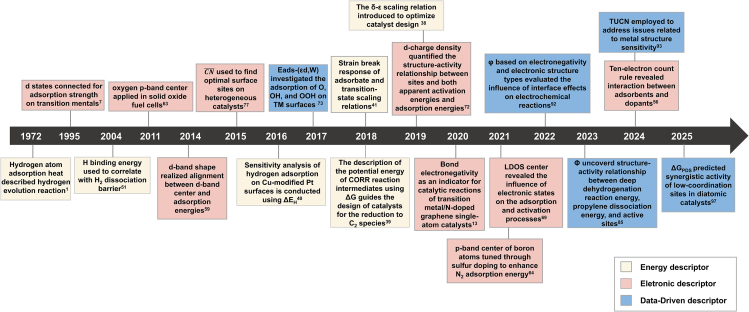
Timeline of the development of electronic descriptors

The use of descriptors in designing optimal catalytic active sites has been widely studied. This review traces the evolution of these descriptors, from early energy-based models focused on adsorption heat and used to describe the hydrogen evolution reaction, to more advanced electronic and data-driven approaches (Figure 1). We explore how these descriptors provide insights into molecular adsorption characteristics, reaction intermediate states, and reaction barriers. Additionally, we highlight their application in designing single-atom catalysts, dual-atom catalysts, nanoalloy catalysts, organic catalyst and novel catalytic sites. The incorporation of these descriptors has significantly accelerated the development of innovative catalysts and paved the way for the discovery of new materials.

## Types and characteristics of electronic descriptors

### Energy descriptors

**Figure 2:**
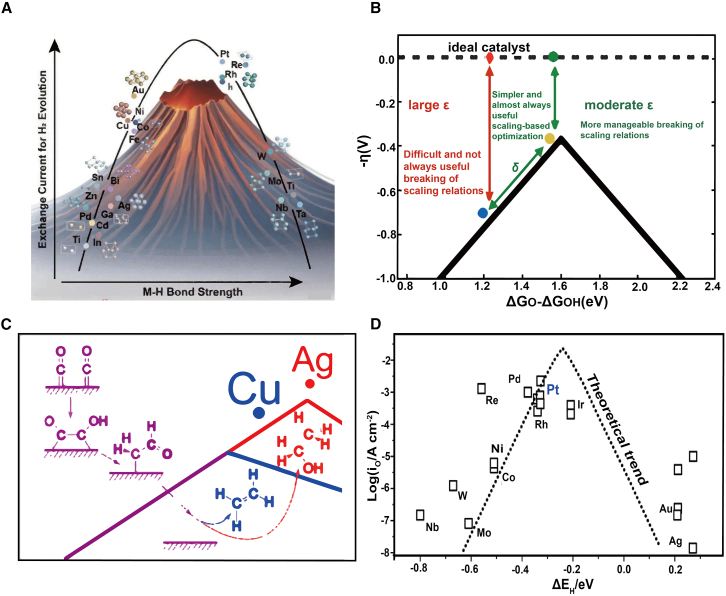
Energy descriptors in electrocatalytic reactions (A) Depiction of the HER using adsorption heat. The catalyst demonstrates optimal activity when the hydrogen atom adsorption energy is approximately 55 kcal/mol.^1^ (B) Comparison between δ-ε optimization and extreme ε optimization. The δ-ε optimization provides a quantitative assessment of the ease of optimization for each catalyst and specific reaction steps. (B) Reproduced with permission from ref.^21^. Copyright 2019 American Chemical Society. (C) Conversion of CO into various C_2_ products on different transition metal (100) surfaces. At low overpotentials, Cu (100) produces ethylene, whereas Ag (100) forms ethanol. (C) Reproduced with permission from ref.^22^. Copyright 2018 American Chemical Society. (D) Trends in the hydrogen evolution reaction. The use of ΔE_H_ as a descriptor effectively predicts the optimal active sites for the HER reaction. (D) Reproduced with permission from ref.^23^. Copyright 2016 Springer Nature.

Energy descriptors are key tools for predicting active sites in catalytic reactions by analyzing the Gibbs free energy or binding energy of reaction intermediates. In 1972, Trasatti introduced the hydrogen atom adsorption on various metals as a descriptor for the hydrogen evolution reaction (HER) (Figure 2A), demonstrating that optimal catalyst activity occurs when the adsorption energy reaches approximately 55 kcal/mol.^1^ This work marked the beginning of using descriptors for designing highly efficient electrocatalytic materials and established a fundamental relationship between catalyst activity and adsorption energy.

Trasatti’s findings also spurred further research into other electrocatalytic reactions. In 2004, Nørskov et al. advanced this concept by developing a method to calculate the stability of reaction intermediates in electrochemical processes using electronic structure calculations.^24^^,^^25^ This approach accounted for alternative reaction mechanisms involving proton/electron transfer to adsorbed molecular oxygen, improving the electrocatalytic performance of fuel cell cathodes.^25^ For energy descriptors, adsorption energy became a key factor, revealing a “scaling” relationship between the adsorption free energies of surface intermediates. This relationship is expressed as follows:

(Equation 1) $$\begin{array}{c} \Delta G_{2}^{j}=A*\Delta G_{1}^{j}+B \end{array}$$

where A and B are constants dependent on the geometric configuration of the adsorbate or adsorption site.^26^^,^^27^^,^^28^^,^^29^^,^^30^ This “scaling” relationship not only simplified material design but also highlighted inherent limitations in electrocatalytic efficiency. Notably, not all catalytic systems follow a linear relationship. In various metal reaction sites, a linear connection exists between dissociation activation energy and chemisorption free energy, known as the Brønsted-Evans-Polanyi (BEP) relationship.^31^^,^^32^^,^^33^^,^^34^^,^^35^^,^^36^^,^^37^^,^^38^^,^^39^^,^^40^ Both adsorption energy and transition state energy in catalytic reactions are strongly influenced by this relationship, which limits the ability of energy descriptors to fully capture the electronic properties of metal surfaces. Overcoming these limitations remains a key challenge in designing optimal catalytic active sites.

Many studies have focused on breaking the scaling relationships between reaction intermediates to design more efficient catalysts. However, achieving the ideal conditions to reduce overpotentials remains a challenge. In this context, tensile strain may influence binding energies, thereby breaking the scaling relationships of catalysts.^41^ To address this, researchers have turned to descriptor-based analysis (DBA) methods to predict electrocatalytic activity. For example, in the oxygen evolution reaction (OER), two independent parameters (Figure 2B), δ (limited by adsorption energy scaling) and ε (unaffected by scaling relationships), have been introduced to optimize catalyst design and significantly reduce overpotentials.^32^ Breaking proportional relationships between intermediates is crucial for improving electrocatalytic efficiency, though only a few materials meet these criteria.

The selective of appropriate descriptors is governed by several factors, including electrolyte composition (ion concentration, pH), solvent properties (dielectric constant, donor/acceptor number), interfacial electric fields, and the electronic structure of the system. In acidic media, nonspecific anion adsorption (e.g., ClO_4_^−^, CH_3_SO_3_^−^) can disrupt the reversibility of the ∗O↔∗OH transition on Pt (111), thereby impeding kinetics and reducing ORR activity.^42^ Consequently, anion concentration emerges as a critical external descriptor influencing ∗O↔∗OH transition rates. In alkaline electrolyte, while the hydrogen binding energy (ΔG_H_) remains a reliable descriptor for the HER, the hydroxyl binding energy (ΔG_OH_) shows a weak correlation with catalytic activity; This indicates that OH^−^ does not directly participate in the rate-determining step but may instead affect ∗H stability through indirect interactions or competitive adsorption, rather than serving as an independent descriptor. The applicability of specific descriptors is pH-dependent, with ΔG_H_ being more appropriate under alkaline conditions.^43^ Additionally, solvation and interfacial interactions regulate the relevance of molecular descriptors (e.g., ESP and band structure) in the design of battery materials.^44^^,^^45^ Therefore, a comprehensive descriptor framework must account for external-field effects on surface adsorption, electronic structure, and reaction kinetics to ensure accuracy and transferability.

Energy descriptors can assess electrocatalytic performance across different reactions, providing a theoretical foundation for designing high-performance catalysts. Logadottir et al. demonstrated that the BEP relationship could predict nitrogen adsorption enthalpy in ammonia synthesis,^34^ whereas the binding energies of intermediates such as ΔG_∗C2O2_ and ΔG_∗OH_ have been shown to forecast CO reduction (CORR) trends on various metal surfaces (Figure 2C).^22^ Additionally, copper monolayers or submonolayers on platinum can modulate hydrogen adsorption energy, enhancing HER activity (Figure 2D).^23^ These strategies not only optimize reactions but also offer practical guidance for reducing the costs of electrolyzers and fuel cells.^46^^,^^47^^,^^48^^,^^49^^,^^50^^,^^51^

### Electronic descriptors

#### Band center theory

**Figure 3:**
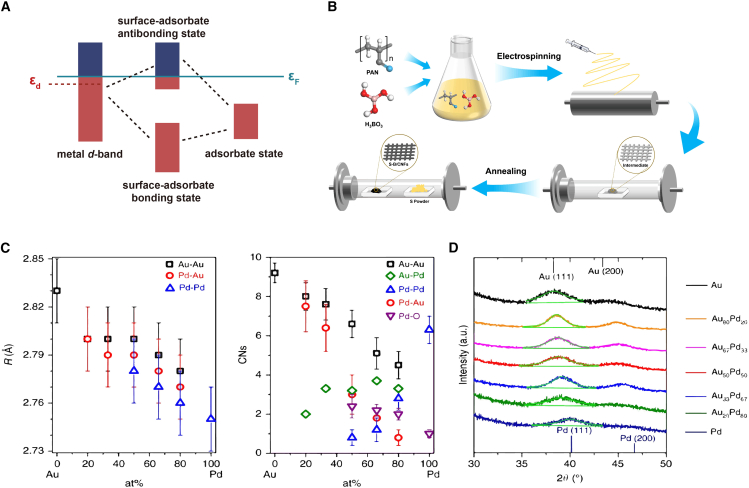
Representative electronic descriptors in metal catalysts (A) Schematic of bonding interactions on transition metal surfaces. A lower the *d*-band center energy relative to the Fermi level results in more filled the antibonding states, leading to weaker adsorption bonds. (B) Relationship between the *p*-band center of S-B/CNFs hybrid materials and NRR activity. (C) Fourier transform EXAFS fitting results for bond distance (R) and coordination numbers (CNs) of AuPd nanoalloys and monometallic nanoparticles. Pd is atomically dispersed within Au, whereas Au tends to aggregate into islands. (D) Fine scan *d*-WAXRD pattern around the diffraction peak at approximately 39°, which includes higher-order statistical data. The symmetrical diffraction peak at around 39° indicates the presence of nanoalloys rather than a core-shell structure of monometallic elements. (C and D) Reproduced with permission from ref.^53^. Copyright 2019 Springer Nature.

The band center, representing the average energy level of specific orbitals, serves as a critical descriptor for understanding electronic structures at catalytic sites.^52^ Among these, the *d*-band center is widely used to analyze the electronic structure of metal surfaces. Introduced by J.K. Nørskov and Bjørk Hammer, the *d*-band center theory established a groundbreaking correlation between the position of the *d*-band center relative to the Fermi level and adsorption strength.^52^ For transition metals, the total electronic band structure can be divided into *sp*, *d*, and other bands. The reconstructed orbitals formed by the *2p* orbitals and *sp* bands have similar energy ranges and shapes, whereas the *d*-band plays a crucial role, as the energy of *d*-band relative to the Fermi level predicts bond strength. Higher *d*-band center energies generally lead to stronger adsorbate bonding due to elevated anti-bonding state energies (Figure 3A), a phenomenon confirmed by X-ray emission and absorption spectra.^7^^,^^52^ However, catalysts with low *d*-state energies often fill anti-bonding states, weakening adsorption bonds. While the *d*-band center effectively correlates adsorption energies for many transition metal surfaces, it struggles with systems where reaction kinetics outweigh thermodynamics, such as strongly correlated oxides. Despite its limitations, the *d*-band center remains a cornerstone in understanding how metal surfaces interact with adsorbates, aiding in the rational design of efficient catalysts. This descriptor is typically calculated using DFT by analyzing the density of states (DOS) for the *d*-orbitals. Mathematically, it is expressed as follows:

(Equation 2) $$\begin{array}{c} \varepsilon_{d}=\frac{\int E\rho_{d}\left(E\right)dE}{\int \rho_{d}\left(E\right)dE} \end{array}$$

where *E* is the energy relative to the Fermi level, and $\rho_{d}\left(E\right)$ is the DOS of the *d*-orbitals at energy *E*.

Under the influence of a magnetic field, abrupt changes in ground-state electronic configurations can lead to piecewise-linear behavior in key electronic properties such as electronegativity (χ) and hardness (η). Geerlings and Proft found that carbon exhibits distinctly different χ and η slopes at magnetic field strengths of B = 0.2 B_0_ versus 0.6 B_0_, owing to spin-state-dependent variations in orbital occupation.^54^ Magnetic-field-induced spin polarization further splits the 3*d*-band into spin-resolved components (ε_d↑_, ε_d↓_), thereby extending the conventional *d*-band-center model. By incorporating spin-dependent hybridization into the Newns-Anderson model, one can accurately reproduce the linear trends observed in adsorption energies (e.g., NH_3_ on ferromagnetic Fe or Co).^55^ To capture the role of spin polarization, an effective *d*-band center can be defined as follows:

(Equation 3) $$\begin{array}{c} \varepsilon^{eff}=\sum_{\sigma }\frac{f_{\sigma }\varepsilon_{d\sigma }}{\sum_{\sigma }f_{\sigma }}-\left(\varepsilon_{d↓}-\varepsilon_{d↑}\right)\mu \end{array}$$

where $f_{\sigma }$ denotes spin-resolved occupancy, and μ is a normalization factor that encapsulates the impact of spin polarization on electron filling. Spin splitting reshapes the *d*-state density: on ferromagnetic surfaces the minority-spin band shifts closer to the Fermi level, increasing antibonding occupancy and thereby weakening adsorption. Conversely, the opposite trend is observed on antiferromagnetic surfaces.

In magnetic bimetallic systems such as NiPt and FePt, traditional linear adsorption scaling relations often breaks down. By introducing surface magnetization ($m_{surf}$) in conjunction with the ab initio *d*-band center ($\varepsilon_{d}^{slab}$), a new spin-sensitive scaling descriptor emerges. SISSO (Sure Independence Screening and Sparsifying Operator) analysis reveals that the adsorption energy slope correlates with $\varepsilon_{d}^{slab}\left(m_{surf}^{2}-1\right)$.^56^ Accordingly, for a given adsorbate Y, the adsorption energy can be expressed as follows:

(Equation 4) $$\begin{array}{c} {\Delta E}_{Y}={\Delta E}_{O}\left[c_{Y}\varepsilon_{d}^{slab}\left(m_{surf}^{2}-1\right)\right]+\beta_{Y} \end{array}$$

where $c_{Y}$ quantifies the extent to which $m_{surf}$ amplifies or suppresses adsorption strength. This spin-resolved, magnetization-inclusive framework transcends the limitations of conventional valence-band scaling, providing a new paradigm for the rational design of high-performance ferromagnetic catalysts.

Building on the *d*-band model, Schumann et al. introduced the “ten-electron counting rule,” providing a molecular orbital approach to catalytic activity.^57^ This rule states that optimal adsorbate binding on dopant atoms in single-atom alloy surfaces occurs when the total valence electrons of the metal dopant (υ_M_, corresponding to its group number in the periodic table) and the adsorbate (k) equal 10 (υ_M_ + k = 10). This approach complements the *d*-band model, offering enhanced predictive power for catalytic performance, particularly for single-atom catalysts. Traditionally, catalytic performance has been described by binding energy, as emphasized by the Sabatier principle, which highlights the direct link between binding energy and catalytic activity, rooted in the electronic structure of the catalyst. Although the *d*-band center theory has been pivotal in explaining catalytic behavior, its applicability to single-atom catalysts is limited due to the localized electronic structure of single atoms and its inability to account for strong metal-support interactions. The ten-electron counting rule, employing a molecular orbital approach, offers a powerful alternative. It has proven effective in identifying promising catalysts for industrial hydrogenation reactions, significantly narrowing the pool of potential materials by more than an order of magnitude. This approach not only complements the *d*-band model but also provides deeper insight into binding trends at reactive dopant sites, advancing the rational design of highly efficient catalysts.

Metal oxides exhibit intricate electronic structures, making it challenging to establish clear correlations between the binding energies of OER intermediates (∗OH, ∗O, ∗OOH) and tunable material properties. In reducible oxides such as TiO_2_, oxygen vacancies introduce excess electrons that directly modulate intermediate adsorption energies by the octet rule. ∗OH and ∗OOH each requires one electron to complete an eight-electron shell, whereas ∗O requires two. Consequently, the number of excess electrons (NEE) dictates an intermediate’s electron-capture capacity.^8^ On these surfaces, NEE follows a volcano-type correlation with OER activity and aligns closely with the conventional ΔG_O_–ΔG_OH_ descriptor. Leveraging this insight, Mo-decorated TiO_2_ (110) is predicted to achieve a theoretical overpotential of 0.54 eV, offering a cost-effective, non-noble alternative to Ru/Ir catalysts and a new strategy for OER catalyst design. Moreover, the *d*-band center—quantifying metal-site electronic levels and filling at metal sites—provides a direct “structure-adsorption-activity” linkage and serves as a universal descriptor across diverse materials classes (two-dimensional [2D] supports, metal-organic frameworks [MOFs], covalent-organic frameworks [COFs]). Together, these advances pave the way for a transition from trial-and-error to data-driven paradigms in the rational design of efficient catalysts for HER, ORR/OER, and CO_2_R.

Despite its utility, the *d*-band center often fails to account for variations in alloy systems, even when considering the effects of *d*-band width^58^ and *sp*-electrons.^59^ To address these shortcomings, the *d* band edge ($\varepsilon_{d}^{w}$) was introduced.^60^ Defined as the highest peak in the Hilbert transform of the DOS projected onto the *d*-orbitals, ε_u_ incorporates both the average energy and dispersion of *d*-states. Alternatively expressed as $\varepsilon_{d}^{w}$ = ε_d_ + W_d_/2 (where W_d_ is the *d*-band width), this measure provides a more comprehensive representation of the electronic structure. The *d*-band edge is particularly effective in predicting the reactivity of late transition metals and their alloys, as it accounts for the influence of both energy and dispersion on anti-bonding states. By correlating ε_u_ with the adsorption energies of simple adsorbates and their hydrides,^26^^,^^61^ it enables the prediction of surface reaction energies at locally perturbed metal sites. This capability is invaluable in identifying and optimizing alloy catalysts for enhanced performance. These techniques—ultraviolet photoelectron spectroscopy (UPS), angle-resolved X-ray photoelectron spectroscopy (ARPES), and X-ray photoelectron spectroscopy (XPS)—are essential for probing surface electronic energy distributions and density of states, thereby enabling precise experimental determination of the *d*-band center.^62^ However, the reliability of the *d*-band edge diminishes under certain conditions. For instance, systems with wide *d*-orbitals or significant electronegativity differences, particularly those involving strongly interacting adsorbates like F, Cl, or OH,^26^^,^^59^ pose challenges. In such cases, complex adsorbate-metal interactions reduce the descriptor’s predictive accuracy, underscoring the need for complementary approaches to capture these nuanced effects.

In systems without *d*-orbitals, the *p*-band center offers an alternative descriptor. It refers to the position of the *p*_*z*_ orbital center in heteroatoms, such as boron or sulfur, within carbon-doped materials. For solid oxide fuel cell (SOFC) cathode materials, the oxygen *p*-band center (defined as the bulk oxygen *p*-orbital energy relative to the Fermi level) and the oxygen vacancy formation energy represent effective descriptors of oxygen reduction reaction (ORR) activity.^63^ Wen et al. found that sulfur doping shifted the *p*-band center of boron atoms away from the Fermi level, enhancing electron density at adsorption sites and improving nitrogen reduction reaction (NRR) performance.^64^ Experimental studies on sulfur-carbon nanofiber materials have demonstrated their effectiveness, achieving high ammonia yields and Faradaic efficiencies (Figure 3B).^64^ Use XPS and EELS to analyze elemental chemical states and electronic structure, verifying the impact of doping on B-atom *p*-orbitals. Similar shifts in the *p*-band center of metal oxide layers predict zinc ion adsorption energy and diffusion barriers, aiding in the design of protective zinc anodes.^65^ However, improper tuning of the *p*-band center may increase energy barriers, reducing catalytic performance and highlighting the need for careful optimization.

Jiao et al. demonstrated that the valence *p*-band peak position (E_p_) correlates linearly with the hydrogen adsorption energy (ΔG_H_).^66^ In N-doped graphene, varying the heteroatom (e.g., N vs. B) shifts E_p_ and thereby optimizes hydrogen binding. Whereas the *d*-band center is a powerful descriptor for transition-metal catalysts, *p*-band centers are more suitable for metal-free systems. In oxide catalysts, tuning the O 2*p*-band center through cation choice, coordination environment, and doping or strain engineering enables precise control over intermediate adsorption strength.^67^ *In situ* X-ray absorption spectroscopy can track dynamic shifts in the O 2*p*-center during reaction, and when combined with DFT and data-driven screening, this approach accelerates the discovery of high-activity catalysts for OER, CO_2_R, and beyond.

In many metal oxides, strong hybridization between O 2*p* and metal *d* orbitals produces a valence band dominated by O 2*p* states and a conduction band dominated by metal *d* states, resulting in a semiconductor, like band gap. Unlike a single *d*-band center, which cannot account for ligand contributions or charge-transfer effects, the band gap directly reflects the ligand-to-metal charge-transfer energy. This gap can be experimentally measured via DRUV-Vis absorption edges and quantified using the Kubelka-Munk function or DFT calculations. Experimentally, the activation energy for propene to acrolein oxidation scales linearly with the catalyst’s band gap at reaction temperature: smaller gaps yield lower barriers and faster rates. The band gap thus serves as a proxy for the effective metal *d* orbital energy and corresponding adsorption strength. For example, substituting V into Mo oxides lowers the conduction-band minimum (placing V 3*d* below Mo 4*d*), which enhances O-H bond formation and optimizes the overall oxidation pathway.^68^

Localized electronic states, such as the *d*-states of transition metals or *f*-states of lanthanides, form discrete energy levels that couple with continuous energy bands like the *p*-band. The local density of states (LDOS) center provides insight into how electronic states at specific sites impact adsorption processes.^69^^,^^70^ Defined as

(Equation 5) $$\begin{array}{c} \varepsilon =\frac{\int_{-\infty }^{\infty }\varepsilon \rho \left(\varepsilon -\varepsilon_{0}\right)d\varepsilon }{\int_{-\infty }^{\infty }\rho \left(\varepsilon -\varepsilon_{0}\right)d\varepsilon } \end{array}$$

where *ρ* is the local density of states (LDOS), *ε* is the energy, and *ε*_0_ is a reference constant, typically the Fermi level, the LDOS-center reflects the average position of energy bands. However, it does not directly capture electron migration potential, necessitating complementary analyses. Computational studies suggest that the LDOS center of the Bi 6*p* orbital on the BiOBr (001) surface lies near the vacuum level, indicating strong electron migration capability.^70^ Although band center theories offer simplified insights into electronic structures, they often rely on single-electron approximations that overlook electron-electron interactions, limiting their predictive accuracy. To date, most research has focused on metal sites, highlighting the need to extend these methodologies to non-metal systems for broader applicability.

#### Orbital charge theory

Orbital charge theory provides an electron-based descriptor that links active site properties to activation or adsorption energies by quantifying the charge in unoccupied *d*- or *p*-orbitals. The electronic and adsorption characteristics of alloys are strongly influenced by their structural configuration, such as eutectic or core-shell arrangements. Expanding on *d*-band theory, Professor Ying Wan introduced *d*-charge density as a descriptor of catalytic performance, determined through X-ray absorption near edge structure (XANES) spectroscopy.^53^^,^^71^ XANES spectra capture the white line peak of metal atoms, with the intensity of this peak corresponding to the number of unoccupied *d*-electron orbitals. This approach allows for a quantitative evaluation of *d*-charge density (Figure 3C). Experimental findings revealed that alloy compositions containing 33%–50% palladium achieved a 9-fold increase in reaction rate compared to pure palladium, demonstrating the potential of *d*-charge density as a predictive tool for catalytic efficiency. By providing a robust framework for linking experimental observations with catalytic behavior, *d*-charge density represents a valuable approach to descriptor construction from an experimental perspective.

#### Work function

**Figure 4:**
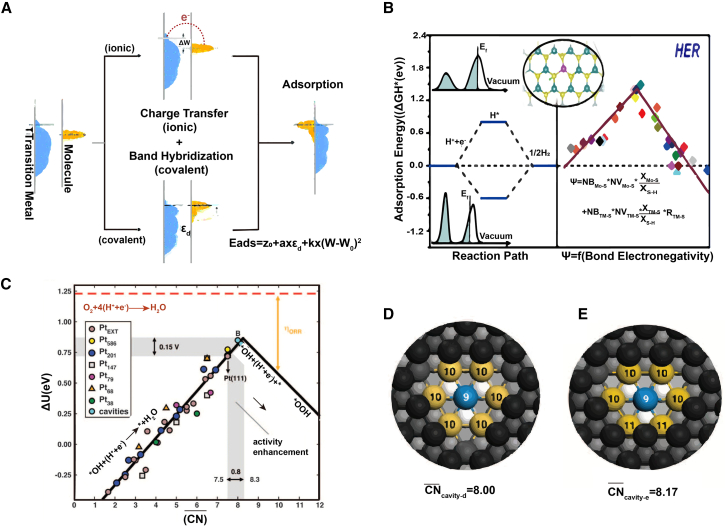
Typical electronic descriptors in metal catalysts (A) Schematic illustrating the ionic and covalent contributions to the adsorption of molecules on transition metal (TM) surfaces. The adsorption energy (E_ads_) decreases as both ionic and covalent bonds contribute to the overall adsorption energy. (A) reproduced with permission from ref.^72^. Copyright 2017 Royal Society of Chemistry. (B) Bond electronegativity Ψ describing HER electrocatalytic activity. The descriptor Ψ shows a linear correlation with ΔG_H∗_. (B) Reproduced with permission from ref.^13^. Copyright 2020 American Chemical Society. (C) Coordination-activity plot. The optimal catalytic activity site has an average coordination number $\bar{CN}$ = 8.3. (D) Top view of a six-atom cavity on Pt (111) with an average coordination number $\bar{CN}$ = 8. (E) Top view of a five-atom cavity on Pt (111) with an average coordination number $\bar{CN}$ = 8.17. (C–E) Reproduced with permission from ref.^74^. Copyright 2015 American Association for the Advancement of Science.

The work function, or escape work function, represents the minimum energy required to remove an electron from a metal’s surface. This parameter has gained recognition as a novel electronic descriptor, building on the principles of *d*-band center theory.^72^ Although the linear correlation between adsorption energy (E_ads_) and the *d*-band center (ε_*d*_), as proposed by Hammer and Nørskov, effectively explains hydrogen and carbon monoxide adsorption,^52^^,^^73^ it does not fully capture the complexities of other adsorption systems. Since the adsorption energies of reactants, intermediates, and products play a critical role in determining the catalytic performance of transition metals (TMs),^72^ researchers have introduced a multi-parameter model. This approach combines the work function (W) with ionic and covalent interaction descriptors to better predict TM activity, particularly in oxygen reduction reactions (ORR) (Figure 4A). The work function is mathematically defined as the difference between the vacuum energy level (*E*_v_) and the Fermi energy level (*E*_f_):

(Equation 6) $$\begin{array}{c} W=E_{V}-E_{f} \end{array}$$

For adsorbates, the Fermi level corresponds to the highest occupied molecular orbital (HOMO), making the work function W = ∣E_HOMO_∣. By incorporating the work function into adsorption models, researchers achieve a more nuanced understanding of adsorption processes and improve the accuracy of catalytic performance predictions. This expanded framework offers valuable insights for the rational design of transition metal catalysts.

An external electric field can modulate a material’s work function by altering its carrier density and shifting the Fermi level, thereby tuning its surface electronic states. In graphene, the work function W_sample_ is defined as the difference between the vacuum level E_vac_ and the Fermi level E_F_, with additional corrections for adsorbates. By applying a back-gate voltage Vg, one adjusts the carrier density and shifts E_F_ relative to the charge-neutrality point (CNP, at Vg = V_D_).^75^ Electron doping raises E_F_ and lowers W_sample_, whereas hole doping lowers E_F_ and raises W_sample_. This continuous tunability arises from graphene’s linear band structure. Experimentally, scanning Kelvin-probe microscopy measures the contact-potential difference ΔV_CPD_ and via eΔV_CPD_ = W_elec_–W_sample_ (W_elec_ is the metal electrode work function), directly quantifies ΔW_sample_.

#### Electronegativity

Electronegativity, which measures an atom’s ability to attract bonding electrons, serves as an essential electronic descriptor linking catalytic activity to electron transfer capabilities.^76^ This property encompasses contributions from both gap-state and atomic valence electrons. Atoms with higher electronegativity exhibit a stronger tendency to attract electrons, a trait that is instrumental in predicting chemical bond behavior, polarity, and reactivity. This descriptor has also facilitated the establishment of universal design principles. For instance, the catalytic activity of transition metal disulfides in the HER is strongly correlated with the ability of active sites to transfer electrons (Figure 4B).^13^ Building on this, Xu et al. combined coordination number with electronegativity to elucidate the electrocatalytic mechanisms of single-atom electrocatalysts, particularly in HER, oxygen evolution reaction (OER), and oxygen reduction reaction (ORR) systems. Their study focused on transition metal/N co-doped graphene, providing a framework for understanding catalytic processes in these systems.^10^ Furthermore, catalytic activity is strongly influenced by local bonding characteristics, including valence electron count, bond electronegativity, and bond distance. These insights have been particularly valuable in designing novel HER catalysts, including various MoS_2_ structures and analogous materials, highlighting the versatility of electronegativity as a tool for rational catalyst design.

#### Generalized coordination number theory

The generalized coordination number (GCN), also known as the mean coordination number, provides a theoretical framework to link changes in the coordination environment of active sites with variations in energy during adsorption reactions.^74^ It is calculated by summing the coordination numbers of the neighboring atoms at an active site and dividing by the maximum coordination number to yield an average value.^77^ This calculation is expressed by the following equation (Equation 7):

(Equation 7) $$\begin{array}{c} \bar{CN\;}\left(i\right)=\sum_{j=1}^{n_{i}}\frac{cn\left(j\right)}{{cn}_{\max}} \end{array}$$

Here, *cn*(*j*) represents the coordination number of each nearest neighbor, and *cn*_max_ is the maximum coordination number for the site. When all neighboring atoms have identical coordination numbers (*cn(j)* = *cn*_max_), the average coordination number ($\bar{CN}$) equals the conventional coordination number (cn), as shown (Equation 8):

(Equation 8) $$\begin{array}{c} \bar{CN\;}\left(i\right)=\sum_{j=1}^{n_{i}}\frac{{cn}_{\max}}{{cn}_{\max}}=n_{i}=cn\left(i\right) \end{array}$$

Although Nørskov’s *d*-band center theory effectively described the reactivity of late transition metals, it struggled to predict the projected density of states (*p*DOS) for early transition metals, fully filled *d*-band metals, and strongly correlated systems.^78^ To address these limitations, DFT simulations have been applied to nanoparticles featuring diverse facets, edges, and corners.^79^

In this context, Calle-Vallejo et al. introduced the GCN as a geometric descriptor to correlate adsorption energy and reaction energy barriers with metal atom coordination numbers. They created catalytic sites with precisely controlled coordination numbers via electrochemical surface modification and then used cyclic voltammetry to measure adsorption behavior and ORR activity, thereby demonstrating the predictive power of the average coordination number.^74^ Their findings revealed that lower coordination numbers lead to stronger molecular adsorption, yielding more accurate linear trends than traditional descriptors.^80^ This approach improves our understanding of nanocatalyst adsorption behavior and offers a geometric framework for designing pure metal catalytic sites (Figures 4C–4E). This theory has been effectively applied to metal systems by relating adsorption activation energy to the coordination numbers of surrounding atoms. Its application to doped and non-metallic systems remains limited and requires further exploration.

### Data-driven descriptors

Conventional descriptors—typically confined to a handful of phenomenological parameters such as metal identity, *d*-electron count, or ligand electronegativity—often fail to capture complex internal architectures, multifaceted electronic interactions, and synergistic effects across different material classes. Machine learning addresses these shortcomings by automating both model selection and hyperparameter optimization within meta-learning frameworks (e.g., Auto MatRegressor), thereby minimizing labor-intensive trial-and-error.^81^ A rigorous data-governance pipeline—comprising SISSO or LASSO for feature selection, GAN-based data augmentation, and the integration of domain knowledge—ensures high-quality inputs, prunes redundant descriptors, and mitigates overfitting in low-data regimes.^82^ Concurrently, generative models (e.g., GAN, VGE) can propose entirely new candidate structures, vastly expanding the searchable materials space.^83^ Looking forward, the convergence of quantum computing and artificial intelligence is poised to reveal quantum-mechanical effects in catalysis and further solidify machine learning as a cornerstone of materials-genome engineering. In essence, data-driven descriptors distill the most salient features of complex systems and establish robust quantitative structure-property relationships, thereby bridging theoretical computation, experimental characterization, and practical application.

#### Isolation degree descriptor (Φ)

The isolation degree is an electronic descriptor designed to characterize the microenvironment of active sites in single-atom alloys (SAAs). It quantifies how isolated the active metal atoms are from their neighboring atoms—a critical factor influencing catalytic activity and selectivity.^84^ Geometric isolation often enables single-atom alloys to achieve high catalytic efficiency and selectivity. However, interference from neighboring atoms, both geometric and electronic, can obscure the definition and performance of these active sites.^85^^,^^86^ To address this, Chang et al.’s research group proposed the isolation degree (Φ), which evaluates the microenvironment and effectiveness of active sites in SAAs.^84^ Φ is defined as (Equation 9):

(Equation 9) $$\begin{array}{c} \Phi =\frac{\frac{r_{M}}{r_{p_{t}}}\Delta \chi \Delta d}{n} \end{array}$$

**Figure 5:**
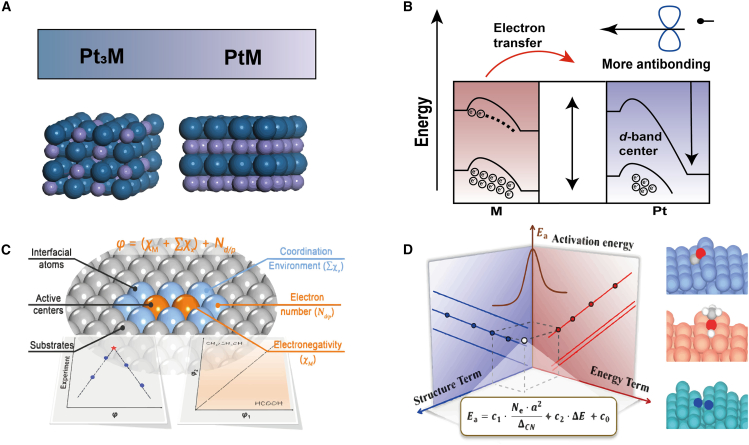
Data-driven descriptors in metal catalysts (A) Close-packed surfaces of Pt_3_M (111) and PtM (110), where blue represents Pt and purple represents M. (B) Schematic of electron transfer from M to Pt, leading to a downward shift of the Pt *d*-band center, which results in more anti-bonding occupation during interaction with adsorbates. (C) The universal descriptor φ, composed of inherent atomic properties (electronegativity [χ], electron types, and numbers [N_d/p_]), which can effectively evaluate the complex effects on the activity and selectivity of CO_2_ reduction reactions. (C) Reproduced with permission from ref.^87^. Copyright 2022 American Chemical Society. (D) Topological under-coordination number mediated by valence electron count and lattice constant. The two-dimensional descriptor, consisting of structural and reaction energy terms, shows extremely high accuracy in predicting reaction barriers and explaining the structural sensitivity of metals. (D) Reproduced with permission from ref.^88^. Copyright 2024 American Chemical Society.

Here, r_M_ and r_Pt_ are the atomic radii of the active metal *M* and platinum (Pt), respectively; Δχ is the electronegativity difference between *M* and Pt; Δ*d* represents the distance difference between Pt and its nearest neighboring *M* atoms; and n is the number of electron shells of *M*. $r_{M}$, $r_{Pt}$, and Δd are measured by structural characterization (XRD, HRTEM, and STM), whereas $\chi_{Pt}$ and $\chi_{M}$ are obtained from standard chemical tables without experimental measurement. This descriptor has been shown to effectively predict the catalytic performance of single-site alloy catalysts. For instance, the experimentally synthesized PtMn single-site alloy catalyst achieved over 93% propylene selectivity in propane dehydrogenation reactions, attributed to its high isolation degree (Figures 5A and 5B).^84^ While promising, the isolation degree descriptor is primarily applicable to single-site alloy systems. Its relevance to other catalyst types, such as supported catalysts, remains limited, necessitating further exploration to broaden its utility.

#### A universal structural descriptor (σ)

A universal structural descriptor, σ, was introduced to quantify the influence of axial ligands (ACLs) on single-atom catalysts (ACL-SACs). It combines the metal’s valence electron count ($\theta_{v}$), its electronegativity ($E_{M}$), and a Hund’s-rule correction ($a_{Hund}$). Qiao et al. calculated adsorption free energies (ΔG) of over 300 oxygen intermediates (OH∗, O∗, and OOH∗) on 10 ACL-modified 3*d*/4*d*/5*d* transition-metal SACs and found that ΔG correlates strongly with θ_v_, E_m_, and two ligand parameters (direct-ligand electronegativity $E_{L_{1}}$ and the product of indirect-ligand count (n) and their electronegativity·$E_{L_{2}}$).^89^ Systematic analysis of local geometries and intrinsic factors yielded (Equation 10):

(Equation 10) $$\begin{array}{c} \sigma =\frac{a\left(19-2\theta_{v}\right)+a_{Hund}}{bE_{M}}-\left(0.3E_{L_{1}}+0.15nE_{L_{2}}\right)=\sigma_{SAC}+\sigma_{ACL} \end{array}$$

where a = b = 1 are calibration constants; $E_{M}$, $E_{L_{1}}$, and $E_{L_{2}}$ are the electronegativities of the central metal, direct-ligand, and indirect-ligand, respectively; n is the number of indirect-ligand atoms (from XRD/STM/TEM); $\theta_{v}$ and $E_{M}$ are the metal’s valence electrons and electronegativity; and $a_{Hund}$ is obtained from XPS, ultraviolet-visible spectroscopy (UV-Vis), or electron paramagnetic resonance (EPR) measurements. We observe a linear negative correlation between σ and ΔG—larger σ values correspond to stronger adsorption. The ligand-specific term $\sigma_{ACL}=0.3E_{L_{1}}+0.15nE_{L_{2}}$ is inherently negative and increases in magnitude with ligand electronegativity and electron-withdrawing power, thereby weakening adsorption. By linking σ and $\sigma_{ACL}$ to the “structure-adsorption energy-catalytic activity-rate-limiting step” pathway, this descriptor framework enables end-to-end, theory-driven design and performance prediction of high-efficiency electrocatalysts.

#### Multi-factor descriptor (*φ*)

The multi-factor weighted fitting descriptor, *φ*, integrates atomic properties such as electronegativity and atomic radius to quantify the structure-activity relationship between active sites and reaction energy during adsorption activation. Supported catalysts often exhibit exceptional catalytic performance across various reactions; however, traditional descriptors like the *d*-band center^60^^,^^90^^,^^91^ or metal valence states^92^ struggle to address the complex interfacial effects present in these systems. To address this challenge, Ren et al. applied gradient boosting regression (GBR) to pinpoint the *d*-electron count as a critical variable and subsequently formulated φ—a straightforward, universal descriptor rooted in intrinsic atomic properties (electronegativity, electron type, and electron count) (Figure 5C).^87^ Designed for 2D material-supported dual-atom catalysts (DACs@2D) in electrochemical reduction reactions such as CO_2_RR, NRR, and ORR, φ is defined as follows:

(Equation 11) $$\begin{array}{c} \varphi =\left(\chi_{M}+\sum \chi_{x}\right)+N_{\frac{d}{p}} \end{array}$$

Here, $\chi_{M}$ represents the metal atom’s electronegativity, reflecting its electron affinity; $\sum \chi_{x}$ is the sum of electronegativities of support atoms, indicating their influence on the metal atom; and $N_{\frac{d}{p}}$ denotes the number of *d*- and *p*-electrons of the metal atom, capturing its electronic structure. The $N_{\frac{d}{p}}$ is obtained by combining spectroscopies measurements (XPS and UPS) with tabulated atomic electronic configurations. Crystallographic and microscopic techniques (XRD, STM, and HRTEM) are used to determine the identity and coordination number of neighboring atom, whose electronegativities are summed to give $\sum \chi_{x}$. Developed using first-principles calculations, orbital symmetry conservation, and machine learning feature engineering, *φ* effectively characterizes metal-support interactions and quantifies interfacial effects. This approach provides a valuable framework for identifying high-performance catalysts. The reliance on computational tools limits its applicability to more complex systems, such as multi-metal or doped catalysts.

#### Topological undercoordination number

The topological undercoordination number (TUCN) is a structural descriptor developed using multi-task symbolic regression (SISSO) and comprehensive first-principles datasets.^88^ It quantifies the degree of undercoordination of exposed metal atoms, incorporating the influence of valence electrons and lattice constants to capture the structural sensitivity of metal catalysts. Designing efficient heterogeneous catalysts requires a precise understanding of active site behavior. Traditionally, this has been approached through the Brønsted-Evans-Polanyi (BEP) relationship, which correlates the activation energy (Ea) of elementary reactions with the reaction energy (ΔE).^34^^,^^35^^,^^93^^,^^94^ Although effective for similar molecules on different metal surfaces, the BEP relationship neglects geometric and compositional variations, limiting its applicability to more complex catalytic systems. To overcome this limitation, Professors Ouyang Runhai and Li Weixue introduced the TUCN (ΔCN) descriptor (Figure 5D),^88^ which integrates geometric structure sensitivity mediated by valence electrons and lattice constants. The TUCN is defined as (Equation 12):

(Equation 12) $$\begin{array}{c} \Delta \text{CN}=\frac{a^{2}}{A}\sum_{i=1}^{n}\left(1-\sqrt{\frac{N_{s}^{i}}{N_{B}}}\right) \end{array}$$

Here, n is the number of exposed atoms within the surface area A of the unit cell, $N_{s}^{i\;}$ is the coordination number (CN) of the *i*-th exposed atom, $N_{B}$ is the bulk coordination number, and *a*a is the lattice constant. Experimentally, ΔCN is obtained by combining lattice constants from XRD, surface coordination numbers measured via HRTEM or STM, and crystallographic calculations that relate the actual surface area to bulk coordination. The TUCN descriptor provides an effective tool for understanding the structure sensitivity of metal catalysts, particularly in systems where traditional descriptors fall short. Combined with multi-factor weighted fitting electronic descriptors, it enhances the ability to predict active site behavior in catalytic reactions.

#### Multi-atom synergistic descriptor (φ′)

The multi-atom synergistic descriptor, φ′, integrates metal electronic properties, coordination parameters, and periodic-table position to guide multi-factor design of 2D materials. Single-atom catalysts (SACs) derive their activity not only from the central metal atom but also from interactions with neighboring heteroatoms (e.g., N and C), which single-parameter descriptors (such as *d*-band center) fail to capture. Xu et al. evaluated the adsorption free energies of H∗, OH∗, and OOH∗ for HER, ORR, and OER across 112 graphene-supported SACs. Starting from 16 candidate features, Pearson correlation analysis narrowed the set to eight independent factors.^10^ An extreme-gradient-boosting regressor (XGBR) then identified the *d*-orbital valence electron count ($\theta_{d}$) and the atom’s period number (L) as the dominant predictors. Consequently, they defined a multi-atom cooperative descriptor φ′ (for OH∗ adsorption in ORR):

(Equation 13) $$\begin{array}{c} \varphi_{OH}^{\prime }=\alpha_{g}\theta_{d}\times \frac{E_{M}+\frac{1}{L-1}\left(n_{N}\times E_{N}+n_{C}\times E_{C}\right)}{E_{O}} \end{array}$$

Here, $E_{M}$, $E_{N}$, $E_{C}$, and $E_{O}$ are the electronegativities of the transition-metal center, first-neighbor nitrogen, carbon and oxygen atoms, respectively; $n_{N}$ and $n_{C}$ are the coordination numbers of N and C atoms around the metal center; L is metal’s period; and $\alpha_{g}$ is a fitting constant. The valence-electron count, $\theta_{d}$, is inferred from XPS-derived oxidation states, whereas neighbor identity and coordination are obtained from EXAFS. A linear regression of φ′ against ΔG_OH∗_ (or ΔG_H∗_) enables rapid, high-throughput screening of SACs candidates (e.g., Fe-pyridine/pyrrole-4N for ORR, Co-pyrrole-4N for OER, and Y-pyrrole-4N for HER), thus overcoming the “one reaction-one descriptor” constraint.

#### Quantitative activity descriptor (ϕ)

Single-atom doping can tune electronic structure and adsorption energy, yet its effect on catalytic activity lacks fundamental understanding and efficient prediction. To address this, a universal descriptor ϕ was derived from a combination of DFT screening and machine-learning techniques. In 2D MXene HER study of 27 single-atom-doped Ti_2_CO_2_ MXenes (across 3*d*, 4*d*, and 5*d* metals) and 81 distinct H-adsorption sites, DFT identified trends in ΔG_H_ and conductivity enhancements.^95^ Kernel ridge regression (KRR) feature selection reduced an initial pool of 18 descriptors to two (Fermi level $E_{f}$ and the geometric distortion parameter $d_{M_{1}-O}$), which sufficed for accurate predictions without overfitting. Symbolic regression (SR-GP) then yielded:

(Equation 14) $$\begin{array}{c} \phi =\frac{E_{f}}{d_{M_{1}-O}-r_{M_{1}}} \end{array}$$

where $E_{f}$ is the Fermi level (an electronic driving force for H adsorption, with higher values implying better electron mobility), $d_{M_{1}-O}$ is the bond length between the dopant M_1_ (Ti, Zr, and Ta) and oxygen in the MXene lattice, and $r_{M_{1}}$ is the covalent radius. The denominator thus quantifies the local geometric distortion induced by single-atom doping: smaller $d_{M_{1}-O}-r_{M_{1}}$ values correspond to stronger M-O bond contraction, which promotes electronic rearrangement at the H-binding site. Using ϕ to screen candidates identified W-doped Ti_2_CO_2_ as optimal: W incorporation triggers a semiconductor-to-metal transition, significantly increases the Fermi-level density of states, and strengthens the H-O bond (ICOHP rising from 1.978 to 2.357 eV), in excellent agreement with the descriptor’s prediction. This high-throughput ML synergy bridges data-driven screening and physical interpretation, offering a universal framework for 2D catalyst design.

#### Multiscale descriptor (ΔG_PDS_)

The multiscale descriptor ΔG_PDS_ delivers a unified “structure-electronic-performance” model by combining ionization energy (IE), atomic radius (R), and electronegativity ($e_{Neg}$) to rationalize both the high activity of low-coordination sites and the long-range synergistic effects in diatomic catalysts. Fourteen primary features were initially extracted at two levels: (1) the active metal center (atomic/covalent radius, electronegativity, ionization energy, and electron affinity) and (2) its coordination environment (coordination number, ligand identity, charge-density contrast, etc.). An AdaBoost analysis highlighted metal electronegativity ($e_{Neg}^{TM}$, 59.84%) and alloy stabilization energy (E_m_, 13.75%) as the most important descriptors; within low-coordination regimes, electron affinity (E_a_, 57.20%) dominated, whereas in high-coordination environments, E_m_ (62.77%) prevailed.^96^ SISSO then combined these features into an explicit predictive expression:

(Equation 15) $$\begin{array}{c} {\Delta G}_{PDS}=-\frac{1.721\frac{{IE}_{1}^{TM}}{{Em}^{TM}}}{R^{TM}\times e_{Neg}^{TM}}+0.248 \end{array}$$

Here, $e_{Neg}^{TM}$ is computed on the Pauling scale from bond-dissociation energies; IE is measured by UPS on gas-phase atoms; E_m_ is derived from alloy formation enthalpies; and R is obtained from high-resolution STEM-EELS measurements. Applied to the nitrogen reduction reaction (NRR), ΔG_PDS_ explains why low-coordination metal sites both facilitate N_2_ activation and suppress the competing hydrogen evolution reaction. This “feature definition-selection-validation” workflow yields an interpretable, quantitative framework for catalyst design and underscores the pivotal role of coordination environment in tuning descriptor importance.

Energy descriptors, with their characteristic volcano-type relationships, can be evaluated swiftly through conventional DFT calculations; however, they offer only a coarse view, omitting the catalyst’s intricate electronic details. In contrast, electronic descriptors (e.g., *d*-band center, orbital charges) delve into these subtleties, enabling the prediction activity and selectivity trends and offering greater transferability across diverse material classes. Yet, when applied in isolation, they frequently fail to capture the full complexity of catalytic performance. To address these shortcomings, data-driven descriptors harness extensive datasets to reveal concealed structure-property correlations and synthesize multiple physical parameters, thereby delivering markedly improved predictive power. A comparative overview of the advantages and limitations of these three descriptor families is provided in Table 1.

**Table 1:** Comparison of different types of descriptors

Types	Name	Equation	Advantages	Disadvantages	Application scenarios	References
Energy descriptor	ΔG_∗OH_, ΔG_∗OOH_ …...	–	quantitative prediction	scaling-relation limitation, lacks electronic detail	HER, OER ……	Trasatti^1^; Fajín et al.^32^
Electronic descriptor	d band center	$\varepsilon_{d}=\frac{\int E\rho_{d}\left(E\right)dE}{\int \rho_{d}\left(E\right)dE}$	correlates adsorption strength	fails to strongly correlated systems, limited to transition metals	adsorption-energy prediction (transition-metal surfaces)	Hammer and Norskov^7^
	orbital charge theory	–	experimentally quantifiable	require complex spectroscopy	applicable to alloys	Zhu et al.^53^
	work function	$W=E_{V}-E_{f}$	integrates multi-parameter models	require precise vacuum measurements	ORR optimization	Shen et al.^72^
	electronegativity	–	predicts bond polarity	ignore environment effects	single-atom catalyst design	Ran et al.^13^
	generalized coordination number	$\bar{CN\;}\left(i\right)=\overset{ni}{\underset{j=1}{\sum }}\frac{cn\left(j\right)}{{cn}_{\max}}$	quantifies geometric structure, Strong correlation with adsorption energy	ignores electronic structure, limited to metallic systems	metal nanocatalysts	Calle-Vallejo et al.^74^
Data-driven descriptor	isolation degree descriptor	$\Phi =\frac{\frac{r_{M}}{r_{p_{t}}}\Delta \chi \Delta d}{n}$	quantifies atomic isolation,	not suitable for supported catalysts	single-atom alloy catalysts	Chang et al.^84^
	a universal structural descriptor	$\sigma =\frac{a\left(19-2\theta_{v}\right)+a_{Hund}}{bE_{M}}-\left(0.3E_{L_{1}}+0.15nE_{L_{2}}\right)$	quantifies ligand effects, applicable to various transition metals	limited applicability to ligand-free systems	ligand-containing single-atom catalysts	Qiao et al.^89^
	multi-factor descriptor	$\varphi =\left(\chi_{M}+\sum \chi_{x}\right)+N_{\frac{d}{p}}$	compatible with machine learning feature engineering	poor applicability to multi-metal or doped systems	2D-material-supported dual-atom catalysts	Xu et al.^97^
	topological undercoordination number	$\Delta \text{CN}=\frac{a^{2}}{A}\sum_{i=1}^{n}\left(1-\sqrt{\frac{N_{s}^{i}}{N_{B}}}\right)$	quantifies undercoordination degree	ignores electronic state details	structure-sensitive metal surface catalysis	Shu et al.^88^
	multi-atom synergistic descriptor	$\varphi_{OH}^{\prime }=\alpha_{g}\theta_{d}\times \frac{E_{M}+\frac{1}{L-1}\left(n_{N}\times E_{N}+n_{C}\times E_{C}\right)}{E_{O}}$	captures metal–neighbor synergy	requires multiple electronegativity values	2D-material-supported single-atom catalysts	Xu et al.^10^
	quantitative activity	$\phi =\frac{E_{f}}{d_{M_{1}-O}-r_{M_{1}}}$	captures geometric distortion effects, accounts for multiple adsorbates	parameter sensitivity prone to errors	2D MXene single-atom-doped HER	Wang et al.^95^
	multiscale descriptor	${\Delta G}_{PDS}=-\frac{1.721\frac{{IE}_{1}^{TM}}{{Em}^{TM}}}{R^{TM}\times e_{Neg}^{TM}}+0.248$	unified structure–electronic–performance framework	complex parameter acquisition	mainly limited to diatomic catalysts	Cheng et al.^96^

## Application of descriptors in active site design

Descriptors play a pivotal role in the design of single-atom, dual-atom, nano-alloy, and organic catalytic sites by effectively predicting catalytic activity and selectivity based on electronic interactions. Beyond considering the composition and structure of catalysts, descriptors also account for surface site interactions, enabling more accurate identification of optimal active sites. The continued development of descriptors has introduced innovative methodologies and provided deeper insights into catalytic processes. These advancements not only enhance our understanding of catalyst behavior but also open new avenues for designing next-generation catalysts with improved performance and selectivity.

### Single-atom catalytic sites

SACs have emerged as a central focus in heterogeneous catalysis due to their high surface free energy,^98^^,^^99^^,^^100^ which enhances activity and selectivity in various chemical reactions. In SACs, active sites consist of isolated single atoms dispersed on a support, promoting strong support-adsorbate interactions and enabling precise control over catalytic reactivity. However, their activity and stability remain significant challenges. SACs are prone to agglomeration or detachment, leading to performance loss, particularly in complex electrochemical reactions. Stability, therefore, is critical, necessitating an in-depth understanding of reaction pathways and intermediates.

**Figure 6:**
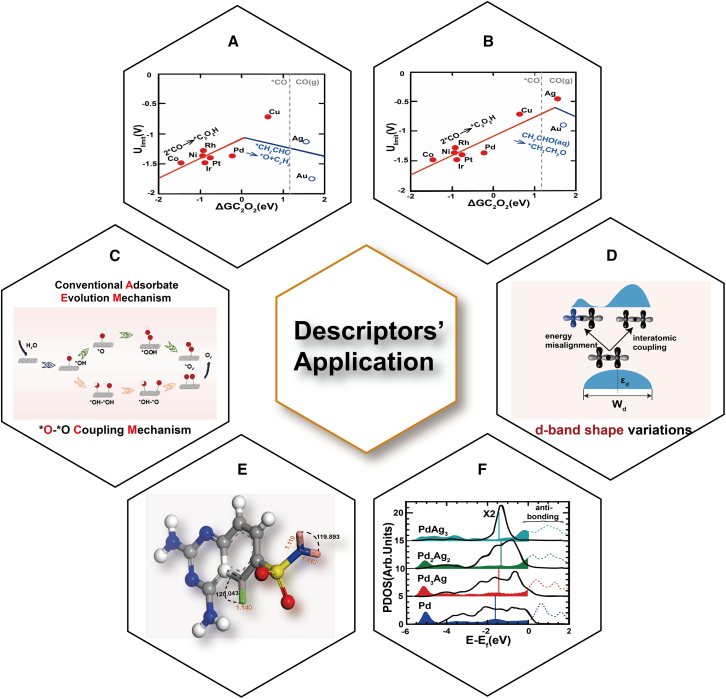
Application of descriptors in catalytic design (A) Volcano plots using ΔG_C2O2_ as a descriptor for ethylene pathways on Cu (100) facets. (B) Volcano plots using ΔG_C2O2_ as a descriptor for ethanol pathways on Ag (100) facets. (A and B) Reproduced with permission from ref.^22^. Copyright 2018 American Chemical Society. (C) Schematic illustration of the possible conventional adsorbate evolution mechanism (AEM) and ∗O-∗O coupling mechanism (OCM). (C) Reproduced with permission from ref.^102^. Copyright 2023 Springer Nature. (D) Schematic illustration of the underlying mechanisms for variations in the *d*-band shape upon formation of alloys. (F) *d*-band center of the surface of Pd and Pd alloys. (D and F) Reproduced with permission from ref.^60^. Copyright 2014 American Chemical Society. (E) Basic structure of 2,4-diamino-6-quinazoline sulfonamides (gray is C, blue is N, white is H, yellow is S, red is O, and pink and green are substituents). (E) Reproduced with permission from ref.^103^. Copyright 2016 Springer US.

To tackle these challenges, researchers have utilized a range of descriptors, including energy descriptors for scaling relationships, *d*-band models,^52^ *d*-shell electron counts,^101^ *p*-band centers,^64^ electronegativity,^13^ and a universal structural descriptor σ to design more efficient SACs. Unlike conventional metal catalysts, SACs can break the scaling relationship of adsorption energies, allowing fine-tuning of intermediate adsorption for improved performance. The traditional scaling relation—relying solely on ∗C_2_O_2_—shows large errors in the weak-binding region, whereas a 2D volcano plot using both ∗C_2_O_2_ and ∗OH descriptors, which accounts for C-O intermediate synergy, reduces the mean error to 0.10 V and more accurately captures complex adsorption behavior. Hanselman et al. demonstrated that ΔG (C_2_O_2_) and ΔG(OH) descriptors effectively predict CO reduction pathways to C_2_ species. Their findings showed that Cu (100) surfaces favor ethylene formation at low overpotentials (Figure 6A), whereas Ag (100) surfaces preferentially produce ethanol (Figure 6B).^22^ Such insights highlight the potential of SACs to selectively optimize intermediate adsorption energies for enhanced activity. In propane dehydrogenation reactions, single-atom alloys (SAAs) also exhibit the ability to break scaling relationships, addressing limitations of traditional metal catalysts.^104^ For traditional Pt_3_M alloys, scaling relations dictate that catalysts with low dehydrogenation barriers (hence high intrinsic activity) also bind propylene too strongly, driving deep dehydrogenation and diminishing selectivity. In these alloys, the C_3_H_5_ intermediate bridges multiple Pt atoms, promoting undesired side reactions. By contrast, dispersing isolated platinum atoms on copper or silver nanoparticles confines adsorption to a single Pt site, significantly weakening C_3_H_5_ binding. DFT calculations revealed that platinum significantly lowers the activation energy barrier for further propylene dehydrogenation, boosting both selectivity and conversion rates. Experimental studies confirmed that platinum-loaded SACs (at just 0.1 wt %) maintain strong activity and stability at elevated temperatures.^105^ Furthermore, the binding energy of metal dopants at atomic vacancies critically influences catalyst activity and durability.^98^^,^^105^

Single-atom alloys, known for their high activity and selectivity, are widely used to promote various chemical reactions.^106^^,^^107^^,^^108^^,^^109^ According to the Sabatier principle, effective catalysis depends on the moderate adsorption energy of key reaction intermediates,^110^^,^^111^^,^^112^ influencing activity and selectivity in specific reactions. Although energy-based descriptors such as adsorption energies are valuable for understanding individual atoms or surface sites, they often fail to capture the overall behavior of the catalyst. In contrast, the outermost *d*-orbital electron count has proven to be a reliable predictor of SAC activity. Studies show that the catalytic activity of transition metals on graphene-like carbon nitride (g-CN) monolayers correlates with the adsorption energies of OH and OCH. The outermost *d*-orbital electron count and metal vaporization enthalpy have been proven effective in designing CO_2_RR sites, identifying promising SACs such as Ni@g-CN, Cu@g-CN, and Co@C_2_N.^101^

Recent studies have also explored the 10-electron counting rule, derived from the *d*-band model, as an effective framework for SAC design.^57^ This rule utilizes a molecular orbital approach to predict active sites. For systems lacking d-orbitals, the p-band center can be used to measure the structure-activity relationship between active sites and adsorption energies, aiding in the design of single-atom catalytic sites. In carbon-based materials, doping of heteroatoms like boron, sulfur, or phosphorus into carbon-based materials adjusts the *p*-band center position (Figure 3B).^64^ This doping induces the formation of BC_3_ structures, promotes N_2_ adsorption at the S-C-B site, and reduces energy barrier, improving NRR activity.^64^ This study highlights the relationship between the *p*-band center of heteroatom-doped carbon catalysts and their NRR activity, paving the way for the development of efficient metal-free electrocatalysts for ammonia synthesis.

Integrating theory-driven insights with data fitting techniques has further accelerated the discovery of high-performance single-atom catalysts. To this end, Qi et al. introduce a composite descriptor, Ψ, which weights bond electronegativity, valence electron count, and bond length, and linear regression to correlate Ψ quantitatively with catalytic activity was employed. This approach successfully guided the design of advanced HER catalysts.^108^ Moreover, these methods have revealed how factors such as electronic structure, dopant crystal phase, nitrogen doping, and periodicity influence catalytic activity, providing valuable principles for optimizing SACs. Axial ligands (ACLs) provide an additional lever to tune SAC activity. By reinforcing the M-O bond, diminishing metal-intermediate adsorption strength, and broadening the metal *d*-band dispersion (thereby reducing bonding d-state occupancy), ACLs can dramatically alter catalytic behavior. For example, in CoN_4_-CH_3_, the CH_3_ ligand disperses Co 3*d* states and weakens OH∗ binding.^89^ The universal descriptor, σ, which captures both metal and ligand contributions, correlates linearly with ΔG for oxygen intermediates, enabling rapid ORR/OER performance prediction for ACL-modified SACs without the need for exhaustive DFT calculations. Its ligand-specific term, $\sigma_{ACL}\text{,}$ quantitatively reflects how different ACLs modulate ΔG, thereby pinpointing the rate-determining step and guiding ACL selection to maximize activity. In summary, the development of robust descriptors, ranging from energy-based relationships to advanced machine learning tools, has significantly advanced the design and understanding of single-atom catalytic sites. These insights pave the way for next-generation catalysts with enhanced activity, selectivity, and stability.

### Dinuclear catalyst active sites

Dinuclear catalysts (DACs) offer unique advantages through the synergistic interactions between two metal atoms, enabling enhanced activity and selectivity in multi-intermediate reactions.^113^^,^^114^ This synergy addresses limitations seen in SACs, whose fixed adsorption patterns restrict flexibility, particularly in complex reactions like the oxygen evolution reaction (OER), which are governed by linear scaling relationships.^115^ By optimizing interactions between intermediates, DACs excel in complex catalytic processes that involve multiple reaction steps, making them highly suitable for such applications.

Descriptors such as energy barriers (ΔG, ΔE), *d*-band centers, *p*-band centers, and electronegativity establish clear connections between the geometric and electronic structures of DAC active sites and their catalytic performance.^87^^,^^102^^,^^116^ These tools guide DAC design by evaluating the energy barriers of intermediates in various electrochemical reactions. In the conventional adsorption-evolution mechanism (AEM) for OER, the adsorption energies of ∗OH, ∗O, and ∗OOH intermediates are constrained by linear scaling relations, yielding a theoretical overpotential minimum of 0.37 V. Bimetallic active sites can bypass universal scaling relationships through the ∗O-∗O coupling mechanism (OCM) (Figure 6C), allowing ∗OH to adsorb on different metal atoms, bypass ∗OOH, and form an ∗OH-∗OH intermediate, facilitating catalytic reactions.^102^ Both overly strong and overly weak ∗OH-OH adsorption (ΔG_OH-∗OH_) can inhibit the ∗O-∗O coupling process. At the same time, the OCM mechanism also aids in screening heteronuclear DACs (e.g., M'M@NC) supported by different metal.^102^ The adsorption energies of various reaction intermediates, combined with their *d*-band and *p*-band centers, can also be used to assess the activity of DAC systems in catalytic reactions.

For both homonuclear and heteronuclear DACs, the *p*-band center of ∗OH can optimize the ∗OH adsorption by adjusting the coordination environment and orbital interactions (e.g., *dz*^*2*^ and *dxz* orbitals) of metal atoms,^116^ enhancing ORR activity. For DACs supported on two-dimensional materials (DACs@2D), interface interactions in electrochemical reactions (e.g., CO_2_, O_2_, and N_2_ reduction reactions) are evaluated using electronegativity and electronic type.^87^ These help evaluate and predict the catalytic performance of DACs@2D in various electrochemical reactions, providing valuable guidance for designing high-performance electrocatalysts.

### Nanostructures and alloy catalytic sites

Metal nanoalloy catalysts exhibit intricate electronic structures and diverse geometric configurations,^117^^,^^118^^,^^119^ offering more flexibility in coordination environments compared to single-atom or intermetallic catalysts.^120^^,^^121^^,^^122^ These variations require precise control over the proportion and arrangement of metal components within the alloy to achieve the desired electronic and geometric properties. As a result, a key focus of current research is how to fine-tune these properties to optimize catalytic performance. To address, researchers have developed various descriptors like the *d*-band center, *d*-orbital charge, work function (W), average coordination number $\left(\bar{CN}\right)$, and isolation degree (Ф) to characterize and predict catalytic performance.^52^^,^^53^^,^^72^^,^^74^^,^^84^ Despite these advances, identifying the most effective descriptor for specific reactions remains an ongoing challenge.

Electronic descriptors, such as the *d*-band center, play a crucial role in designing new alloy catalysts by correlating electronic structure with reaction activity and selectivity. For instance, in methane reforming,^52^ a higher *d*-band center relative to the Fermi level strengthens the bonding between the metal surface and adsorbates. However, because the *d*-band center represents the average energy of *d*-orbitals, it may not fully capture the unoccupied portion of *d*-orbitals in alloys. The *d*-band edge, which reflects changes in the shape of the *d*-band, provides a more accurate descriptor, especially when alloying metals like Pd with Ag or Au, where reactivity is influenced by *d*-band modifications (Figures 6D and 6F).^60^

The work function is another critical descriptor in nanoalloy catalyst design. Combining the *d*-band center with the work function has led to the development of the E_ads_-(ε_d_, W) model,^72^ which accurately predicts the adsorption of key intermediates, such as O, OH, and OOH, on transition metal surfaces (Figure 4A). This model has proven effective in evaluating ORR activity. Additionally, for HER, work function differences in single-layer heterojunction catalysts serve as reliable descriptors, facilitating H_2_ production and advancing the exploration of 2D heterostructures.^123^ Li et al.’s research further demonstrates the use of work functions to predict corrosion resistance, aiding in the rational design of metal alloy catalysts.^124^

Transition metals, with unfilled *d*-orbitals, are efficient catalysts that can adjust their electronic structure by altering *d*-orbital electron filling, thereby modifying catalytic performance. As a result, the *d*-orbital charge serves as a descriptor to reflect adsorption states and catalytic performance. In AuPd nanoalloys, for example, spectroscopic analyses such as XPS and XANES have linked *d*-band electron changes to catalytic entropy change and turnover frequency during benzyl alcohol oxidation.^53^ Alloys with 33–50 at% Pd exhibited a 9-fold increase in reaction rate compared to pure Pd, correlating with a maximum *d*-charge gain (Figures 3C and 3D). Unlike other descriptors, the *d*-orbital charge emphasizes electronic adjustments rather than geometric characteristics, providing unique insights into catalytic performance.

Geometric descriptors also play a vital role in understanding nanoalloy catalyst behaviors. The average coordination number, $\bar{CN}$, correlates strongly with catalytic activity by linking the geometric structure of active sites to reaction energetics, allowing for the design of more active nanoalloy catalysts. For ORR on platinum surfaces,^74^ $\bar{CN}$ demonstrates a robust correlation with oxygen species reaction energies, a principle that can extend to other transition metals, including 3*d* metals (Co, Ni, and Cu), 4*d* metals (Rh, Pd, and Ag), and even 5*d* metals (Ir, Pt, and Au). And the $\bar{CN}$ maintains a low root-mean-square deviation (RMSD) correlation with reaction energies.^27^

However, $\bar{CN}$ primarily reflects neighboring atom counts and may not fully capture interatomic interactions. To address this limitation, the isolation degree (Ф) was introduced to describe interactions within the microenvironment of active sites, such as Pt-C repulsion in propane dehydrogenation catalysts.^84^ Alloys with high isolation degrees exhibit significant changes in selectivity based on active center design. Rationally designed descriptors, including Ф, provide deeper insights into tailoring active sites, enabling the optimization of unit site alloy catalysts for enhanced performance.

### Organic catalytic sites

Designing organic catalytic sites demands precise tuning to enhance efficiency and facilitate drug synthesis. Traditional approaches, often based on empirical rules and trial-and-error experimentation, are time-intensive and costly.^103^^,^^125^ Additionally, developing organic compounds with high catalytic activity and selectivity, particularly for bacterial inhibition, remains a formidable challenge. Recent advances have focused on leveraging electronic descriptors to analyze molecular properties, enabling the design of more effective organic catalysts by introducing targeted substituents.

Catalytic activity in organic molecules is strongly influenced by electronic energy and local electron density. By examining these electronic descriptors and strategically modifying substituents, researchers can create organic compounds with enhanced catalytic performance. Organic molecules are essential in drug synthesis due to their functional groups, which exhibit favorable pharmacokinetic properties, such as efficient tissue and fluid penetration, along with ease of metabolism. Typically, sulfonamide drugs have demonstrated potential in various pharmacological applications, including antimalarial drug design. Enhancing catalytic activity can be achieved by introducing electron-withdrawing groups (e.g., nitro or cyano groups) or by selecting substituents with specific sizes to optimize molecular interactions (Figure 6E).^103^

The integration of machine learning and multivariate regression methods with electronic structure data has provided deeper insights into how electronic configurations influence substituents. These techniques guide the design of new derivatives with improved efficacy, such as antimalarial agents. Descriptors like chemical electronegativity and electrophilicity indices reflect a molecule’s polarization capacity and its tendency to attract electrons. Softer molecules and those with higher electronegativity are more likely to interact with proteins.^125^ These electronic descriptors predict properties such as photostability, redox potential, polarity, and affinity, thereby enabling the design of more promising organic catalytic sites. This approach can contribute to further *in vitro* studies for optimal hit identification and the development of targeted antibacterial drugs.

### Novel catalytic materials

#### Metal oxide materials

Designing reliable descriptor for metal-oxide materials remains challenging due to limited universality, complex coupling of multiple factor, and persistent gaps between theoretical predictions and experiment observations. Electronic-structure descriptors (NEE, O 2*p*-band center, and band gap) have shown strong predictive power for catalytic performance, and their utility is further amplified when integrated with machine-learning methods. In particular, NEE provides a quantitative handle on OER activity in reducible oxides like TiO_2_ by accurately predicting the binding strength of intermediates (∗OH, O, and ∗OOH). Moreover, by tuning NEE, one can reproduce the characteristic volcano-type relationship between ΔG_O_ and ΔG_OH_. This insight led to the prediction that Mo-decorated TiO_2_ (110) can achieve a theoretical overpotential of 0.54 eV, providing a non-noble alternative to Ru/Ir catalysts.^8^ Similarly, tuning the O 2*p*-band center shifts both oxygen-vacancy formation energy and adsorption strengths: as the O 2*p* center approaches the Fermi level, vacancies become easier to form and OER kinetics accelerate, a relationship validated across perovskite and related oxides.^67^ Hybridization between transition-metal *d* bands and O 2*p* states further controls bond strength: CO_2_ adsorption on perovskites scales linearly with the *d*-band center. While in propylene oxidation, smaller gaps (e.g., V-doped Mo oxides) correspond to lower barriers and higher activity.^68^

**Figure 7:**
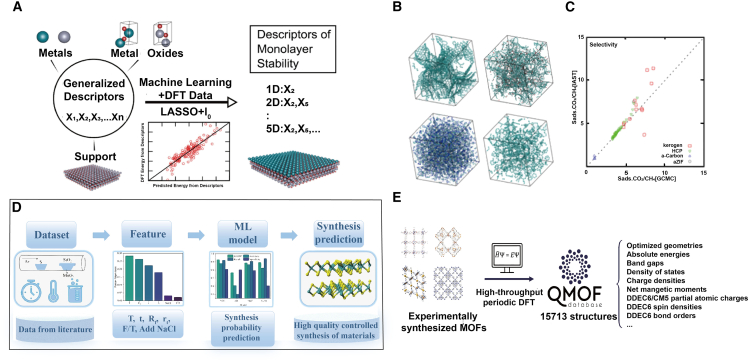
Application of machine learning in new materials design (A) Supervised LASSO + $l_{0}$ regression reveals the key physical descriptors governing MMO stability. For stoichiometric monolayer oxide coatings, stability is dominated by substrate surface energy, orbital radius, and ionization energy; in contrast, the stability of nonstoichiometric coatings depends on the intrinsic stability of the parent oxide and the oxidation-state mismatch at the coating-substrate interface. (A) Reproduced with permission from ref.^126^. Copyright 2018 American Chemical Society. (B) Snapshots of structures contained in the amorphous materials database. (C) Adsorption selectivity (S_ads_) computed from GCMC simulations and IAST for an equimolar bulk mixture at 298 K and total pressure of 10 bar. IAST predictions of CO_2_/CH_4_ selectivity are in good agreement with GCMC simulations, exhibiting a mean absolute percentage error of 8%. (B and C) Reproduced with permission from ref.^127^. Copyright 2020 American Chemical Society. (D) Machine-learning-assisted synthesis of two-dimensional materials. Chemical vapor deposition (CVD) parameters were machine-learning-optimized to achieve efficient, controllable synthesis. (D) reproduced with permission from ref.^128^. Copyright 2022 American Chemical Society. (E) Overview of the QMOF database. Selected DFT-computed properties for the structurally relaxed MOFs made available in the QMOF database. (E) Reproduced with permission from ref.^11^. Copyright 2021 Cell Press.

In fact, conventional single-parameter scaling (solely ΔH_O_) yields RMSE≈0.27eV for OER enthalpies, whereas an SISSO-derived 5D descriptor (combining *d*-band width, charge-transfer energy, and local geometric terms) reduces RMSE≈0.18 eV. Furthermore, LASSO+ $l_{0}$ regression over 1.7 million features reveals that monolayer-oxide coating stability depends jointly on substrate surface energy, orbital radius, and ionization-energy differences (stoichiometric) or on coating-bulk stability and oxidation-state variance (non-stoichiometric) (Figure 7A).^126^

#### Two-dimensional supports

Non-traditional supports (such as 2D materials, MOFs, COFs, and porous polymers) support interactions to achieve high activity, selectivity, and stability. In 2D catalyst design, despite DFT’s fixed-charge approximation and simplified solvent treatment, descriptors like the *d*-band center, valence *p*-band peak position (E_p_), lowest unoccupied state energy ($\varepsilon_{LUS}$), and multifactor local descriptors φ (combining atomic properties and coordination numbers) are widely employed.

For transition-metal sulfides (TMSs) such as MoS_2_ and WS_2_, the metal-edge *d*-band center (*ε*_*d*_) correlates linearly with adsorbate binding strength at S sites, enabling ε_d_ tuning to predict adsorption across different TMSs.^129^ On MoS_2_ basal planes, defect-site *d*-band centers (ε_d_′ within −1 eV–0 eV relative to the Fermi level) more accurately track H-adsorption energies, highlighting how sulfur vacancies enhance H binding. Likewise, the *p*-band center of neighboring S atoms (*ε*_*p*_*′*) governs S-site adsorption linearly, and the valence-band peak (E_p_) guides the design of *p*-orbital-based 2D catalysts (e.g., TM single atoms or B-doped graphene), with higher E_p_ correlating with stronger intermediate (OH∗, OOH∗, and H∗) stabilization and increased activity.^66^

In ORR/OER, Jiao et al. introduced the descriptor E_*diff*_—the difference between the lowest valence-band orbital energy at the active site and the Fermi level—which correlates linearly with ΔG_OH_,^130^ providing a quantitative measure of a material’s electronic structure and its adsorption behavior. In N-doped graphene SACs, the TM *d*-band centers shift with local coordination (e.g., N-dopant number). Notably, for Cu/g-C_3_N_4_, an upward-shifted *d*-band center enhances CO binding and boosts CO_2_-to-C_2_^+^ conversion.^131^ Building on these insights, Xu et al. proposed a DFT-free descriptor φ—based solely on atomic properties and coordination—that predicts H- and O-species adsorption (HER, ORR/OER) with R > 0.94 across 112 SAC models.^10^ Furthermore, Xia and co-workers established a volcano relation from local charge redistribution and heteroatom effects, identifying optimal N-doped graphene active sites for ORR.^132^ Collectively, these descriptor-based strategies—quantifying parameters such as electronegativity, electron affinity, and coordination number—streamline computational screening and furnish clear guidance for the experimental design of next-generation electrocatalysts.

Moreover, machine learning also excels in 2D catalyst systems.^96^^,^^133^ In transition-metal dichalcogenides, alkali-metal adsorption energies scale linearly with the lowest unoccupied state energy ($E_{LUS}$), and ordinary least-squares regression on 112 DFT data points with 6-fold cross-validation achieves high accuracy (R^2^ = 0.968, RMSE = 0.012 eV), with $E_{LUS}$ contributing the most (coefficient = 0.974).^133^ In MXenes (e.g., Ti_2_CO_2_), single-atom dopants tune ΔG_H_; high-throughput DFT + ML identifies universal descriptors linking dopant properties to HER activity.^128^ In MoS_2_ CVD synthesis (Figure 7D), XGBoost, SVM, Naive Bayes, and MLP models pinpoint reaction temperature, Ar flow rate, and time as key parameters; XGBoost optimization yields high-quality nanosheets, showcasing ML’s power to accelerate synthesis and reduce trial and error. Machine learning analyses also identify metal electronegativity ($e_{Neg}^{TM}$) and alloy stabilization energy (E_m_) as critical descriptors for NRR catalyst design.

#### Metal-organic frameworks and covalent-organic frameworks

MOFs and COFs are emerging porous crystalline materials with precisely tunable structures and electronic properties ideal for catalysis. In MOFs, descriptors such as the metal-node *d*-band center, e g-orbital occupancy, and first ionization energy accurately predict OH∗, O∗, and OOH∗ adsorption in line with the Sabatier principle, guiding OER performance design. Moreover, the *d*-electron count of framework metal ions controls electron transfer to Pt nanoparticles: *d*^*0*^*-d*^*4*^ MOFs donate electrons to Pt, enhancing 1-hexene hydrogenation, whereas *d*^*5*^*-d*^*10*^ MOFs withdraw electrons.^134^

By contrast, COFs depend on covalent network-metal coordination interactions. In porphyrin-based COFs, crystal-field stabilization energy (CFSE) and orbital configuration (CE) accurately predict OER/ORR activity: Fe-COF exhibits low overpotentials in the four-electron transfer, rivaling noble-metal catalysts, and (Cu, Zn)-COFs or alkaline-earth COFs (Ca, Sr) are predicted to catalyze H_2_O_2_ production.^12^ These behaviors mirror the control of adsorption energy by the *d*-band center in MOFs.

Traditional descriptors often omit critical variables since electrocatalytic activity arises from nonlinear interactions among metal electronic structure, ligand geometry, and surface charge. Machine-learning-derived descriptors overcome these limitations by mining high-order interactions. DFT relaxations were carried out on 15,713 experimentally synthesized MOFs to build the QMOF database of quantum-chemical properties (band gaps, charge, and spin densities) (Figure 7E). A crystal graph convolutional neural network (CGCNN) was then applied to extract atomic-connectivity features directly from CIF files for rapid band-gap prediction. When combined with unsupervised dimensionality reduction, this framework uncovers structure-property relationships and pinpoints low-band-gap MOFs with computational efficiency orders of magnitude greater than that of conventional DFT.^11^ GNNs can even encode COF interlayer stacking as graph features, automatically identifying structural parameters that govern electron transport and catalytic performance.^135^

#### Porous polymer networks

Porous polymer networks (PPNs) have been computationally designed at scale—17,846 PPNs were generated and optimized by PM6-DH_2_, then screened via grand-canonical Monte Carlo simulations to target CH_4_ storage.^136^ Pore size emerged as the key descriptor, with optimal diameters of 7–10 Å maximizing uptake. This study underscores the importance of pore-size matching and host-guest interactions as guiding principles for experimental synthesis. Thyagarajan and Sholl^127^ constructed an atomic-level database of 205 rigid amorphous porous materials (e.g., PIMs and HCPs), comprising scalar descriptors—pore-limiting diameter (PLD), largest cavity diameter (LCD), and surface area—and vectorial pore-size distributions (PSDs). Grand canonical Monte Carlo (GCMC) simulations yielded single-component and CO_2_/CH_4_ binary adsorption isotherms. Material similarity was quantified by Euclidean distance and a modified Tanimoto coefficient, and ideal adsorbed solution theory (IAST) predictions of binary-adsorption selectivity showed a mean absolute error of 8% (Figures 7B and 7C). The resulting database provides a valuable resource for high-throughput screening and structure-property relationship studies, advancing computational research in gas storage, separation, and related applications.

## Challenges and outlook

**Figure 8:**
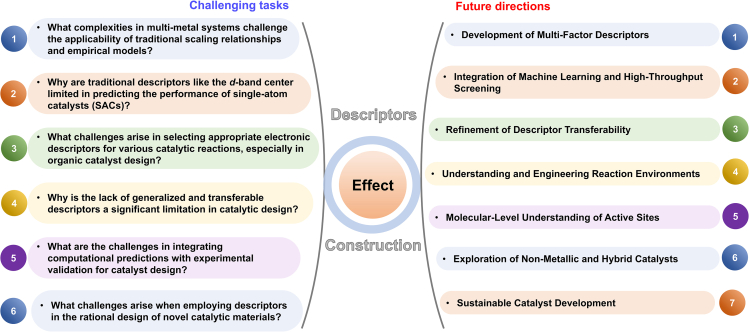
Challenging task and future directions of descriptors construction in catalytic design

The application of descriptors in catalyst design presents both exciting opportunities and substantial challenges. Key descriptors, including the *d*-band center, *p*-band center, *d*-orbital charge, work function, and average coordination number, have significantly advanced the prediction of catalytic performance across SAAs, IMAs, DACs, organic catalytic sites, and novel catalytic materials. However, these tools face limitations in addressing the inherent complexity of multi-metal systems, challenges in optimizing SACs, and difficulties in selecting appropriate descriptors for specific reactions. Traditional scaling relationships and empirical models often fail to capture intricate interactions within multi-metal catalysts, and the rigid active sites of SACs restrict their suitability for multi-step reactions. Similarly, in organic catalyst design, particularly for drug synthesis, the development of effective descriptors remains a critical hurdle for enhancing activity and selectivity (Figure 8).

### Challenges

• Inherent complexity of multi-metal systems

Multi-metal catalysts present significant challenges due to their intricate interactions, dynamic surface restructuring, and non-linear adsorption behaviors. For example, within the conventional AEM mechanism, the adsorption energies of reaction intermediates are constrained by linear scaling relationships. In contrast, bimetallic active sites can overcome these universal scaling limits via the OCM mechanism, facilitating the formation of ∗OH-∗OH intermediates and thereby enhancing the overall catalytic reaction.^102^ Traditional scaling relationships and empirical models often fail to capture these nuanced effects, limiting their predictive power for multi-metal systems.^2^^,^^3^^,^^4^^,^^5^^,^^6^ Accordingly, accurate prediction and rational design demand the development of multifactorial or dynamic descriptors capable of reflecting these intricate effects.

• Limitations in single-atom catalysts

Although SACs have revolutionized catalyst design, their unique localized electronic structures and strong metal-support interactions limit the applicability of traditional descriptors such as the *d*-band center. Current descriptors often fail to capture these effects, making it challenging to fully predict and optimize SAC performance in multi-step reactions.^13^^,^^52^^,^^64^^,^^101^

• Challenges in organic catalyst design

Organic catalytic systems, particularly for drug synthesis, face difficulties in optimizing activity and selectivity due to the lack of effective and transferable descriptors. The incorporation of electronic properties such as electronegativity, polarizability, and electrophilicity into robust models for organic systems remains underexplored.^103^^,^^125^

• Lack of generalized and transferable descriptors

Most descriptors (*d* band center,^52^ *p* band center,^71^ *d* orbital charge,^53^ and so on) are highly system-specific and lack transferability across different catalytic systems and reactions. The absence of universal descriptors hinders the ability to accurately predict activity and selectivity trends for a wide range of catalysts and reaction conditions.

• Integration of experimental and computational insights

Bridging the gap between computational predictions and experimental validation remains a key challenge. The electrolyte environment,^42^ external-field effects,^54^^,^^75^ and electrode-electrolyte interfacial phenomena^44^^,^^45^ can all significantly impact the accuracy of descriptors. Experimental limitations, such as accurately probing the molecular-level structures of active sites and dynamic reaction environments, further complicate the refinement of descriptors.

• Challenges in novel catalytic materials

Novel catalytic materials (metal oxide materials,^8^^,^^67^^,^^68^ 2D supports,^66^^,^^129^^,^^130^ MOFs,^134^ COFs,^12^ and PPNs^127^^,^^136^) exhibit complex electronic structures, and existing descriptors are often tailored to specific systems, limiting their broad applicability. Descriptor-based predictions must be corroborated by experimental data, as many theoretical forecasts still lack sufficient empirical support. Furthermore, dynamic structural changes during catalysis—such as intermediate adsorption configurations—cannot be fully captured by static descriptors alone, necessitating the integration of *in situ* characterization techniques (e.g., DFT) to deepen mechanistic understanding.

### Future directions

Despite these challenges, the field of descriptor-based catalyst design is poised for transformative progress.

• Development of multi-factor descriptors

Combining multiple parameters, such as electronic, geometric, and thermodynamic properties, into comprehensive multi-factor descriptors can provide a more holistic understanding of catalytic systems.^10^^,^^87^^,^^89^ This approach is particularly promising for complex multi-metal systems, SACs and DACs.

• Integration of machine learning and high-throughput screening

Descriptors are evolving from single-system adaptation toward multidimensional intelligent design. Recent advances in machine learning and data-driven methodologies are unlocking unparalleled opportunities to accelerate the discovery of both novel descriptors and high-performance catalysts. For example, MatterGen, a diffusion-based generative model, can produce compositionally diverse, thermodynamically stable inorganic materials.^137^ By incorporating adapter modules, it is possible to impose precise constraints on chemical composition, crystallographic symmetry, magnetization density, band gap, and other key physical properties. Remarkably, MatterGen’s yield of stable new materials (SUN) surpasses previous models by more than 2-fold, whereas its mean structural deviation from DFT-predicted local energy minima is reduced by an order of magnitude. Moreover, the successful synthesis of selected candidates validates the practical potential of this inverse-design approach. At the same time, machine learning algorithms are being applied to mine vast datasets, revealing hidden patterns and correlations that enable rapid, high-throughput screening of complex systems (such as the Fe_35_Ni_29_Co_21_Al_12_Ta_3_ high-entropy alloy^138^), thereby streamlining the identification of promising catalyst compositions.

• Refinement of descriptor transferability

Developing robust, generalizable descriptors that remain predictive across diverse catalytic systems and reaction pathways is essential for advancing rational catalyst design. To this end, researchers have constructed catalytic platforms in which nitrogen-doped graphdiyne derivatives (NGDY) accommodate both single-atom (SAC) and dual-atom (DAC) active sites, thereby closing the loop between DFT simulations, machine learning feature extraction, and experimental validation.^96^ In this framework, the “buffering and low-coordination accumulation” mechanism—initially predicted through combined DFT and ML analyses—is directly confirmed by the superior activity of experimentally synthesized low-coordination catalysts. This synergy not only validates the underlying theory but also paves the way for engineering transferable descriptors with broad applicability across different catalysts and reaction environments.

• Understanding and engineering reaction environments

Engineering the local reaction environment at the catalyst-electrolyte interface presents a promising strategy for enhancing catalytic performance.^42^^,^^43^^,^^44^^,^^45^ This includes optimizing the double-layer structure, solvent dynamics, and ion interactions, particularly for reactions in complex media such as electrochemical systems.

• Molecular-level understanding of active sites

Achieving a molecular-level understanding of active sites, including their dynamic interactions with intermediates and the surrounding environment, is essential for refining existing descriptors and proposing new ones. Multifactor descriptors typically composed of numerous parameters that are not directly accessible via conventional experimental techniques. Instead, *in situ* characterization methods can be employed to quantitatively measure each parameter, thereby enabling the indirect construction of the full descriptor.^10^^,^^89^^,^^97^ Advanced *in situ* characterization techniques, combined with theoretical modeling, will play a critical role in achieving this goal.

• Exploration of non-metallic and hybrid catalysts

The field of non-metallic and hybrid catalysts remains underexplored in terms of descriptor construction. Developing new descriptors tailored for these systems, such as *p*-band centers^71^ or charge-density-based parameters,^53^ could unlock new pathways for catalyst design in sustainable energy and chemical production.

• Sustainable catalyst development

Future descriptors should also consider sustainability metrics, such as cost, scalability, and environmental impact, alongside activity and selectivity. This will be particularly important for industrial-scale applications, including green hydrogen production and CO_2_ reduction.

In conclusion, although significant progress has been made in descriptor-based catalytic site design, addressing these challenges will require an interdisciplinary approach combining computational modeling, experimental validation, and data-driven techniques. The continued refinement and application of descriptors will not only deepen our understanding of catalytic processes but also enable the rational design of more efficient, selective, and sustainable catalysts, driving advancements across energy, environmental, and chemical industries.

## Acknowledgments

The authors thank the Yunnan Fundamental Research Projects (202401AU070180, 202501AT070166) and GuangDong Basic and Applied Basic Research Foundation (2021A1515110427).

## Author contributions

T.L. conceived and coordinated the research. W.Z supervised the project, provided guidance on the structure, and reviewed the final manuscript draft. Y.F drafted the original manuscript and was responsible for visualization, creation, and modification of figures. Y.C. and L.Z. performed the literature search and compiled the data. X.C. provided critical feedback and revised the manuscript. All authors reviewed, edited, and approved the final version of the manuscript.

## Declaration of interests

The authors declare that no competing interests.
